# Absence of SARS-CoV-2 in a captive white-tailed deer population in Alabama, USA

**DOI:** 10.1080/22221751.2022.2090282

**Published:** 2022-06-28

**Authors:** Subarna Barua, Chad H. Newbolt, Stephen S. Ditchkoff, Calvin Johnson, Sarah Zohdy, Rachel Smith, Chengming Wang

**Affiliations:** a College of Veterinary Medicine, Auburn University, Auburn, AL, USA; b College of Forestry and Wildlife Sciences, Auburn University, Auburn, AL, USA

## Main text

While serological and molecular evidence of SARS-CoV-2 infection has been reported in white-tailed deer (*Odocoileus virginianus*) from the USA, deer sera from the UK (*n* = 1748) were found to be negative by a serosurvey. To further understand the geographical distribution of SARS-CoV-2 infected deer, a serosurvey was performed on archived deer serum samples collected from the Auburn University Captive Facility in Camp Hill, Alabama, between October 2019 and January 2022. A surrogate SARS-CoV-2 virus neutralization test identified one positive sample which was later determined to be negative by the virus neutralization testing performed at USDA National Veterinary Services Laboratories. In addition, rectal and nasopharyngeal swabs from deer collected in January and February 2022 were found to be negative by SARS-CoV-2 PCR. Of 72 people who had close contact with the deer over the study period, 29 completed a voluntary questionnaire which showed three had been infected with the SARS-CoV-2 during the study period. Our finding that the deer we studied appeared not to have been exposed to SARS-CoV-2 despite the presence of human infections in the facility indicates spill-over of infections from humans to deer might not be common.

SARS-CoV-2, the agent causing COVID-19 in humans, has been reported to infect domestic (dogs, cats) and wild animals (ferrets, lions, mink, pumas, rodents, snow leopards, tigers) [1]. Recently, infection models demonstrated that white-tailed deer (*Odocoileus virginianus*) were susceptible to SARS-CoV-2 [2–5], and serological and molecular evidence indicated that white-tailed deer from USA (Illinois, Iowa, Michigan, Ohio, Pennsylvania, New York, South California, and Texas) [6–12] and Canada [13] had been exposed to SARS-CoV-2 (Table 1). Vandegrift et al. reported the detection of the highly transmissible SARS-CoV-2 Omicron variant (B.1.1.529) from white-tailed deer, New York [10].

**Table 1.** Molecular and serological prevalence of SARS-CoV-2 in deer

Country, State	Sampling period	Sample; detection method	Prevalence; SARS-CoV-2 lineages identified	Reference
USA: OH	January–March, 2021	Nasal swabs; RT-PCR	35.8%; 129/360; B.1.2, B.1.596, B.1.582	[7]
USA: MI, PA, IL, NY	2011–2021	Serum; sVNT	40.0%; 152/624	[6]
USA: IA	April 2020–January 2021	Retropharyngeal lymph node; RT-PCR	33.2%; 94/283; B.1, B.1.1, B.1.119, B.1.2 51, B.1.234, B.1.240, B.1.264, B.1.311, B.1.362, B.1.400, B.1.459, B.1.596	[8]
USA: NY	December 2021–January 2022	Serum; sVNT	14.5%; 19/131	[10]
		Nasal swabs; RT-PCR	10.3%; 7/68 B.1.1.529	
USA: TX	January–February 2021	Serum; plaque reduction neutralization assay	37.0%; 20/54	[11]
USA: TX	September–November 2021	Serum; plaque reduction neutralization assay	Facility A: 94.4%; 34/36; facility B: 0.0%; 0/16: facility C: 0.0%; 0/29	[9]
		Respiratory and rectal swabs; RT-PCR	0.0%; 0/80	
USA: SC	N/A	Serum; Neutralizing antibody assay	9.0%; 2/22	[12]
Canada: QC	Nov 2021	Nasal swabs; RPLNs; RT-PCR	1.2%; 3/251 (nasal swab); 0.0%; 0/104 (RPLNs); B.1.617.2 (lineage AY.44)	[13]
		Thoracic cavity fluid; neutralizing antibody assay	5.6%; 14/251	
UK	January 2020–May 2021	Serum; sVNT	0.0%; 0/1748	[14]
Germany	January 2020–December 2021	Serum; sVNT	0.0%; 0/181	[15]
Austria	January 2020–December 2021	Serum; sVNT	0.0%; 0/51	[15]

Estimates suggest there may be more than 30 million white-tailed deer in North America, in rural, suburban, and urban areas. They are the most sought after game species in North America with $35M being spent each year by the 11.4 million people who hunt the animals. In Alabama, there are an estimated 1.25 million white-tailed deer, making the human–deer population ratio approximately 4:1. There are about 225,000 licensed deer hunters in Alabama, and Alabama hunters typically harvest approximately 275,000 animals annually over a season that is 109 days in length. The interactions that occur between humans and deer due to hunting and suburban/urban encroachment create a scenario in Alabama where deer could be exposed to SARS-CoV-2. To provide information on SARS-CoV-2 infections of white-tailed deer in a captive facility from the state of Alabama, we carried out a serological and RT-PCR surveys.

Blood samples, and nasopharyngeal and faecal swabs used in this study were collected from deer at the Auburn University Captive Facility in Camp Hill, Alabama. This is a 174-hectare high-fenced facility with a population of approximately 100 deer. Although given ad libitum supplemental feed, these deer are not domesticated and have similar behaviours to free-ranging deer outside of the facility, albeit the latter having more space and a lower population density [16]. In weekdays between October 2019 and February 2022, deer captured after being darted at feeders [16] had body and dental measurements taken and sera and nasopharyngeal swabs collected. This normally took 15–30 min and involved two to five people being in very close contact with the deer and thus creating a high potential for human-to-deer and deer–human-transmission of infectious diseases. The COVID-19 status of the deer handlers at the time of the procedures was obtained from voluntarily completed questionnaires.

All animal procedures were approved by the Auburn University Institutional Animal Care and Use Committee (PRN 2016-2964, PRN 2016-2985, PRN; 2019-3599, PRN 2019-3623). Whole blood was collected into EDTA from the jugulars of 64 deer (seven in October 2019; 25 during 2020; 30 during 2021; two in January 2022) and serum separated and stored at −85°C. In January and February 2022, nasopharyngeal and faecal swabs were obtained from seven deer and stored in 400 µl DNA/RNA stabilization buffer (Roche Life Science) until DNA extraction and PCR as described below.

SARS-CoV-2 Surrogate Virus Neutralization Test (sVNT) Kits were purchased from GenScript (NJ, USA), and used according to the manufacturer’s instructions. The virus neutralization tests (VNT) were performed at the USDA National Veterinary Services Laboratories (NVSL).

The High-Pure PCR Template Preparation Kit (Roche Diagnostics, Indianapolis, IN, USA) was used to extract total nucleic acids from nasopharyngeal and rectal swabs according to the manufacturer’s instructions and described previously [17]. SARS-CoV-2 Reverse-Transcription FRET-PCR was performed as described [18]. Genomic RNA of two SARS-CoV-2 viruses from the American Type Culture Collection (ATCC) (2019-nCOV/USA-WA1/2020; 201/501Y.V1) served as controls and quantitative standards.

In the sVNT, only one sample collected on March 10, 2021 showed an inhibition value of 36.03% and, being above the 30% cut-off value, was regarded as positive. This sample and three others with the next highest inhibition values (20.03%, 18.53%, 17.27%) were submitted to the USDA NVSL for VNT. All four samples were found to be negative in the virus neutralization assay. All nasopharyngeal and faecal swabs were negative for the SARS-CoV-2 by quantitative SARS-CoV-2 PCR. Although we found one sample positive by sVNT, the USDA definition of a confirmed SARS-CoV-2 case in animals requires a positive VNT result, which was not the case with our sample. A variety of coronaviruses such as bovine-like coronaviruses have been identified in cervids in the United States, and there is the possibility that the sVNT-positive deer had been exposed to one of these viruses.

Twenty-nine of the 72 individuals who had been in close contact with the deer during handling and sampling voluntarily completed the questionnaire on their COVID-19 status at the time of contact. Three of the 29 people confirmed they were infected with SARS-CoV-2 during the study period but none reported being in contact with deer either seven days before or seven days after testing positive.

## Discussions

The negative test results for SARS-CoV-2 infections in the deer we studied was contrary to our expectations generated by the high prevalences reported in free-ranging deer [6–10]. The research activities taking place in the Auburn Captive Facility result in relatively high levels of human–deer contact thereby creating favourable opportunities for transmission of SARS-CoV-2 from infected people. This is contrary to the situation for free-ranging white-tailed deer which seldom, if ever, come into close contact with people. Further, contact between deer is greater in our facility than is the case with free-range animals and thus greater prevalences and rates of deer-to-deer transmission of SARS-CoV-2 would be anticipated. The stocking density in the facility is approximately five times greater than that typically seen with free-ranging deer, and our captive deer regularly come into close contact at the three permanent feeding stations in the facility. Data from a captive cervid facility reported by Roundy et al. [9] showed very high rates (94.4%) of SARS-CoV-2 infections which supported our hypothesis that we should have found a high prevalence if the deer in our facility had acquired SARS-CoV-2.

At least three people who worked in our deer facility and came into close contact with the deer were positive for SARS-CoV-2 during the study period, although they reported not having contact with deer seven days before or after testing positive for SARS-CoV-2. The remaining 43 people who came into close contact with the deer did not provide data, but it seems likely some of them would also have been infected with the SARS-CoV-2 and would have had the opportunity to pass the infection to the deer. Wu et al. [19] reported many people are SARS-CoV-2 positive, yet unaware because they were never tested and/or never developed symptoms. They suggest that the number of people infected may be 3–20 times greater than the number of cases that are confirmed through testing [19]. These expected high rates of infection amongst the workers at the deer facility suggest it is possible that our deer were in fact exposed to SARS-CoV-2 positive people but did not become infected. This raises the possibility that SARS-CoV-2 spill-over from humans to deer may be less common than initially suggested by other studies [6–10].

Our finding of unexposed deer is not unique, with no animals being found to be seropositive in a large survey of 1748 deer in the UK [14], and in Germany and Austria [15]. Similarly in Texas, deer in only one of three captive facilities were found to have been infected [9]. The patchy distribution of seropositive deer could indicate that SARS-CoV-2 spill-over from infected humans to deer is low, as indicated by our study. However, while it is generally agreed that deer are directly infected with SARS-CoV-2 from humans, other potential transmission routes such as via rodents and contaminated wastes cannot be excluded. It appears likely, though, from the high seroprevalences seen in deer that once animals are infected there is efficient transmission within a herd. The results of the studies to date indicate there is an urgent need for further active surveillance and longitudinal studies to more completely understand the ecology of SARS-CoV-2 in deer and other animals.
